# Morphology of the dysplastic hip and the relationship with sex and acetabular version

**DOI:** 10.1002/ca.24174

**Published:** 2024-05-08

**Authors:** Inger Mechlenburg, Sepp De Raedt, Hakim C. Achterberg, Maiken Stilling, Lone Rømer, Kjeld Søballe, Marleen de Bruijne

**Affiliations:** ^1^ Department of Clinical Medicine Aarhus University Aarhus Denmark; ^2^ Department of Orthopaedics Aarhus University Hospital Aarhus Denmark; ^3^ Department of Radiology and Nuclear Medicine Erasmus MC Rotterdam Rotterdam The Netherlands; ^4^ Department of Radiology Aarhus University Hospital Aarhus Denmark; ^5^ Department of Computer Science University of Copenhagen Copenhagen Denmark

**Keywords:** hip, hip dysplasia, morphology, sex, three‐dimensional model

## Abstract

The dysplastic hip is characterized by incomplete coverage of the femoral head, resulting in increased risk of early osteoarthritis. The morphological variation of the hip joint is diverse and clear differences exist between females and males. The aim of this observational study was therefore to investigate the relationship between the morphology of the hip, sex, and hip dysplasia using a three‐dimensional model. Statistical shape models of the combined femur and pelvic bones were created from bilateral hips of 75 patients. Using manual angle measurements and regression analysis, the characteristic shape differences associated with sex and hip dysplasia were determined. The model showed clear differences associated with sex and hip dysplasia. We found that the acetabular anteversion in females was significantly higher (*p* < 0.0001) than in males while no significant difference in acetabular anteversion was found between normal and dysplastic hips (*p* = 0.11). The model showed that decreased acetabular anteversion resulted in the appearance of the cross‐over sign and the prominent ischial spine sign commonly associated with retroversion. Sex could be predicted with an area under the curve of 0.99 and hip dysplasia could be predicted with an area under the curve of ≥0.73. Our findings suggest that retroversion is a result of decreased anteversion of the acetabulum and is primarily associated with sex. This finding should be taken into account during the reorientation of the acetabulum in the surgical treatment of hip dysplasia.

## 1 INTRODUCTION

The morphology of the hip joint and its relationship with hip diseases and sex is not well understood. In clinical practice, it is apparent that there is a wide spectrum of acetabular configurations and subtle variations and combinations of different deformities are often observed. In diseases such as hip dysplasia and femoroacetabular impingement syndrome (FAIS), the bone morphology leads to an abnormal biomechanic relationship between the femur and acetabulum causing pain and disability (O'Brien et al., 2023). Young people with hip dysplasia are 5‐times more likely (Wyles et al., 2017) to develop hip osteoarthritis (OA), undergo major hip replacement surgery by age 35 years than those without hip dysplasia (Lodhia et al., 2016) where the average age to undergo hip replacement is 65 years (Ackerman et al., 2019). Having hip replacement at a young age may result in up to three joint replacement revisions over a lifetime, each at higher cost with poorer outcomes (Ackerman et al., 2021). In hip dysplasia, the acetabulum is characterized by a shallow socket with a steep roof resulting in lacking global coverage of the femur (Mechlenburg et al., 2004). In FAIS, with a pincer morphology, the acetabulum is described to be deep with over‐coverage of the femoral head leading to risk of impingement of the labrum (Ganz et al., 2003). In FAIS, with a cam deformity, a bump at the neck–head junction of the proximal femur results in increased risk of impingement with the anterior rim of the acetabulum (Ito et al., 2001). In between the two extremes described by hip dysplasia and FAIS, there are varying degrees and combinations with both extreme and normal morphological variation of the acetabulum and femur (Mechlenburg et al., 2019). Another important morphological variation of the pelvis is the direction of the acetabular opening, which is described by the version of the acetabulum (Mast et al., 2004). On conventional radiographs, the version is judged by examining the shadows of the lateral edge of the anterior and posterior walls (Mast et al., 2004). In an anteverted acetabulum the opening points anteriorly and the posterior wall remains lateral to the anterior wall. In retroverted acetabula, the opening is orientated more posteriorly and the posterior and anterior walls cross‐over forming a figure eight (Mast et al., 2004; Reynolds et al., 1999). Although it is generally thought that hip dysplasia is associated with an anteverted acetabulum, previous work has found that up to one third of dysplastic hips are retroverted (Mast et al., 2004; Tannast et al., 2012). During the surgical treatment of hip dysplasia, it is important to take into account the version of the acetabulum to normalize the anterior and posterior coverage (Bræmer et al., 2020; Mast et al., 2004). It is therefore important to understand how the version of the acetabulum varies with respect to normal and dysplastic hips.

The dimorphism of the pelvis with respect to sex is well established (Decker et al., 2011). Females have a greater angle of the pubic arch, a wider and lower pelvis and a larger pelvic inlet than males. In addition, it is also known that the incidence of symptomatic hip dysplasia is up‐to four times higher in females than in males (Dembic et al., 2023; El Jashi et al., 2017). Conversely, it has also been found that cam impingement is more common in males (Ganz et al., 2003). It is however unclear if the difference in shape of the pelvis may be associated with the difference in incidence of the two diseases. In this work, we therefore aim to obtain a better understanding of the relationship between the shape variation of the hip and sex.

Statistical shape modeling is a common technique that can be used to study the morphological variation and the association with different characteristics (Sarkalkan et al., 2014). Statistical shape models capture the mean shape of a set of shapes and the main modes of variation based on principle component analysis (PCA). In previous work, the two‐dimensional morphology of the femur and acetabulum have been investigated based on AP radiographs. Various studies have used two‐dimensional statistical shape models to predict the development of OA and risk of total hip replacement (Agricola et al., 2013; Barr et al., 2012). Three‐dimensional studies on the proximal femur have investigated the relationship between shape and Legg‐Calvé‐Perthes and FAIS with cam morphology (Chan et al., 2013; Harris et al., 2013). However, no previous work has studied the three‐dimensional shape variation in a combined pelvis and femur model and the association with hip dysplasia and sex. In this study, we therefore aimed to study the association between morphology, sex and hip dysplasia in order to better understand the morphology of the hip. We created combined models of the pelvic bones and femurs and used them to study the association by regression analysis. In the following section we will introduce the process used to construct the statistical shape model and perform shape analysis.

## 2 METHODS

In this retrospective cross‐sectional study, 75 patients (50 females), median age 36 (13–65) years were identified that underwent CT investigation of the hip between January 2006 and October 2008 at Aarhus University Hospital, Denmark. Patients were referred to scanning due to symptomatic hip pain, most commonly due to suspected hip dysplasia. A positive diagnosis of hip dysplasia was defined as a center‐edge (CE) angle less than 25°. Scans were acquired according to a standardized protocol with the patient in a supine position and legs in a neutral position. The scan volume ranged from superior to the acetabulum to approximately below the lesser trochanter.

Scans were acquired on a Philips Mx8000, Philips Brilliance 40, or Philips Brilliance 64 (Philips Medical Systems Best, Best, The Netherlands) scanner. Scan resolution and spacing varied, but the mean voxel size was 0.45 mm × 0.45 mm × 1.25 mm. In‐plane voxel size ranged from 0.38 to 0.52 mm and the out‐of‐plane voxel size ranged from 1.25 to 1.6 mm.

### 2.1 Manual angle measurements

An experienced radiologist (LR) performed manual measurements on a Philips PACS workstation (Philips Medical Systems, Best, Netherlands). The radiologist measured the CE angle, acetabular index (AI), anterior acetabular‐sector angle (AASA), posterior acetabular‐sector angle (PASA), and acetabular anteversion (AcAV) angle were measured according to the standard definition (Mechlenburg et al., 2019). The horizontal acetabular‐sector angle (HASA) was calculated as the sum of the AASA and PASA.

### 2.2 Point correspondence

We used of a statistical shape model commonly referred to as a point distribution model (PDM), which represents shapes as a collection of points and models the mean shape and the variation of the shapes observed in the dataset. The construction of the PDM requires that the points are corresponding across shapes, that is, they determine the same anatomical location in different subjects. We first segmented bone structures from the CT image and then, for each femur and pelvic bone for both left and right hips separately, used non‐rigid group‐wise image and shape registration to establish point correspondences.

Bone structures were segmented using an automatic graph cut segmentation method as described in Raedt et al. (2013). The obtained bone segmentations were dilated and soft‐masks were created by smoothing the distance transform of the binary segmentations.

The group‐wise registration procedure requires the pairwise, deformable registration of each of the bones from all subjects. The individual registrations consisted of a sequence of rigid, affine, and non‐rigid deformations using the CT images and corresponding soft‐masks, as detailed in Online Supplement 3. The resulting pairwise transformations for a subject were subsequently averaged to obtain the mean transformation, which maps points from the individual subject space to the mean space. After thus transforming the soft‐mask to the mean space for each subject, a voxel wise averaging is performed to obtain a mean soft‐mask. The mean shape is then extracted from the mean soft‐mask image using the marching cubes algorithm (Lorensen & Cline, 1987).

To obtain the bone shape for each of the individual subjects, the mean shape is then non‐rigidly transformed to the subject space, using the inverse transform as established by an affine and B‐spline registration with a displacement magnitude metric (Metz et al., 2011).

### 2.3 Shape alignment and pose correction

Due to differences in positioning at the time of scanning, the femur pose with respect to the acetabulum may vary significantly between subjects. Therefore, we performed pose correction of the femurs to a mean pose while preserving the location of the center of the femoral head. First, the combined left and right pelvic bones were aligned iteratively using Generalized Procrustes Analysis (GPA) (Gower, 1975) with translation, rotation, and scaling. This establishes the alignment of the pelvic bones and removes size differences between subjects. The same transformation was then applied to the femur bones and the centers of the individual femoral heads were calculated using sphere fitting. Subsequently, the left and right femurs were separately aligned using only rotations and translations, to remove pose differences of the femur between subjects. The individual femurs were then translated such that the center of the femoral head coincided with the initial center point. The pose correction procedure was repeated until convergence.

### 2.4 Shape analysis

The PDM can be constructed using the set of aligned and pose‐corrected shapes obtained from the previous steps, by computing the mean shape and the principal components, which describe the main modes of shape variation that is present in the dataset. For details of this procedure, see Online Supplement 1.

### 2.5 Statistical analyses

Differences in angle measurements were analyzed by a two‐way mixed effects analysis of variance (ANOVA) model with one within‐subject factor (left and right side) and two between‐subject terms for sex and hip dysplasia with interaction between side and both between‐subject terms. We used regression models to derive the characteristic differences in shape that are associated with outcome variables sex, dysplasia, and angle measurements. This allows us to visualize how a certain shape for instance, the population mean would change to be more/less dysplastic, or which 3D shape changes correlate with an increase/decrease in center‐edge angle. For details of this procedure, see Online Supplement 2.

A *p*‐value of less than 0.05 was found to be statistically significant. All analysis was performed with Stata 13 (StataCorp, College Station, USA).

## 3 RESULTS

Angle measurements are summarized in Table 1. No significant interactions were found between side and the between‐subject factors. No significant difference was found with respect to sex for the CE, AI, and the HASA angles. Meaning that the lateral and total anterior and posterior coverage was similar between males and females. A significant difference with respect to sex was found for the AcAV, AASA, and PASA. Meaning that the version of the acetabulum and the posterior and anterior coverage differed by sex. All angle measurements, except for AcAV were significantly different between non‐dysplastic and dysplastic hips.

**TABLE 1:** Summary statistics for angle measurements (degrees) combined for left and right sides.

Angle	Total (*N* = 74)		Male (*N* = 24)		Female (*N* = 50)		Sex	Dysplasia
	Mean	SD	Mean	SD	Mean	SD	*p*‐value	*p*‐value
CE	20.4	9.4	20.6	10.1	20.4	9.1	0.72	<0.0001
AI	13.5	8.2	13.6	7.7	13.5	8.4	0.59	<0.0001
AcAV	20.1	5.4	16.2	4.1	22.1	5.0	<0.0001	0.11
AASA	49.3	9.0	52.7	8.4	47.7	8.9	0.006	<0.0001
PASA	90.1	8.3	86.0	6.9	92.1	8.2	<0.0001	0.001
HASA	139.5	13.3	138.7	12.6	139.8	13.7	0.38	<0.0001

*Note*: One patient had missing angle measurements and was excluded from analysis. *p*‐values for differences with respect to sex and dysplasia are shown.

Abbreviations: AASA, anterior acetabular‐sector angle; AcAV, acetabular anteversion angle; AI, acetabular index; CE, center‐edge angle; HASA, horizontal acetabular‐sector angle; PASA, posterior acetabular‐sector angle.

### 3.1 Shape variations

**FIGURE 1:**
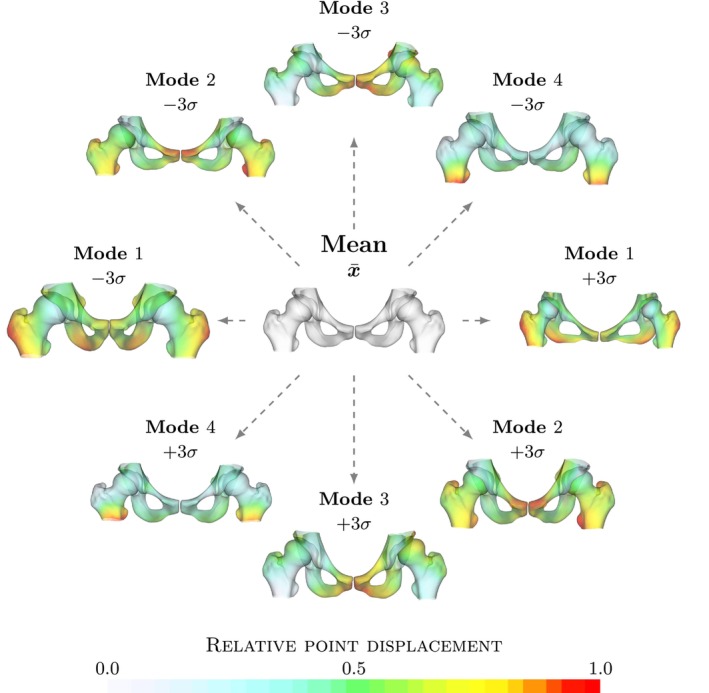
Mean combined pelvis and femur model. Visualization of the mean pelvis and femur model and the four most significant modes explaining 69% of the total variation in the model. Each mode is shown as $\bar{x}$ ± 3 standard deviations. Colors indicate the point displacement normalized by the maximum displacement for a mode. The first four modes of shape variation are illustrated for relative point displacement perturbed for three standard deviation. To facilitate interpretation and highlight differences, the model points are colored by the point displacements, normalized by the maximum point displacement within the mode. Points on the model that move the furthest are indicated by red and points that do not move are gray.

The resulting complete pelvis and femur model is shown in Figure 1. The most predominant difference is in the thickness of the pelvis and in the angle between the pubic bones (pubic arch) (Mode 1). This difference may intuitively be attributed to the difference between sex. The angle is wider in females and forms an inverted u shape while it is acute (<90°) in males as can be seen in Figure 1. A difference in the thickness of the pelvis and femur bones is also visible in the second and third modes. In addition, a clear variation of the coverage of the femoral head by the acetabulum is visible, which is characteristic of hip dysplasia. The transition from a steep roof to a horizontal acetabular roof is especially visible in the third mode. A change in length of the femoral neck and shaft is visible in the fourth mode.

**FIGURE 2:**
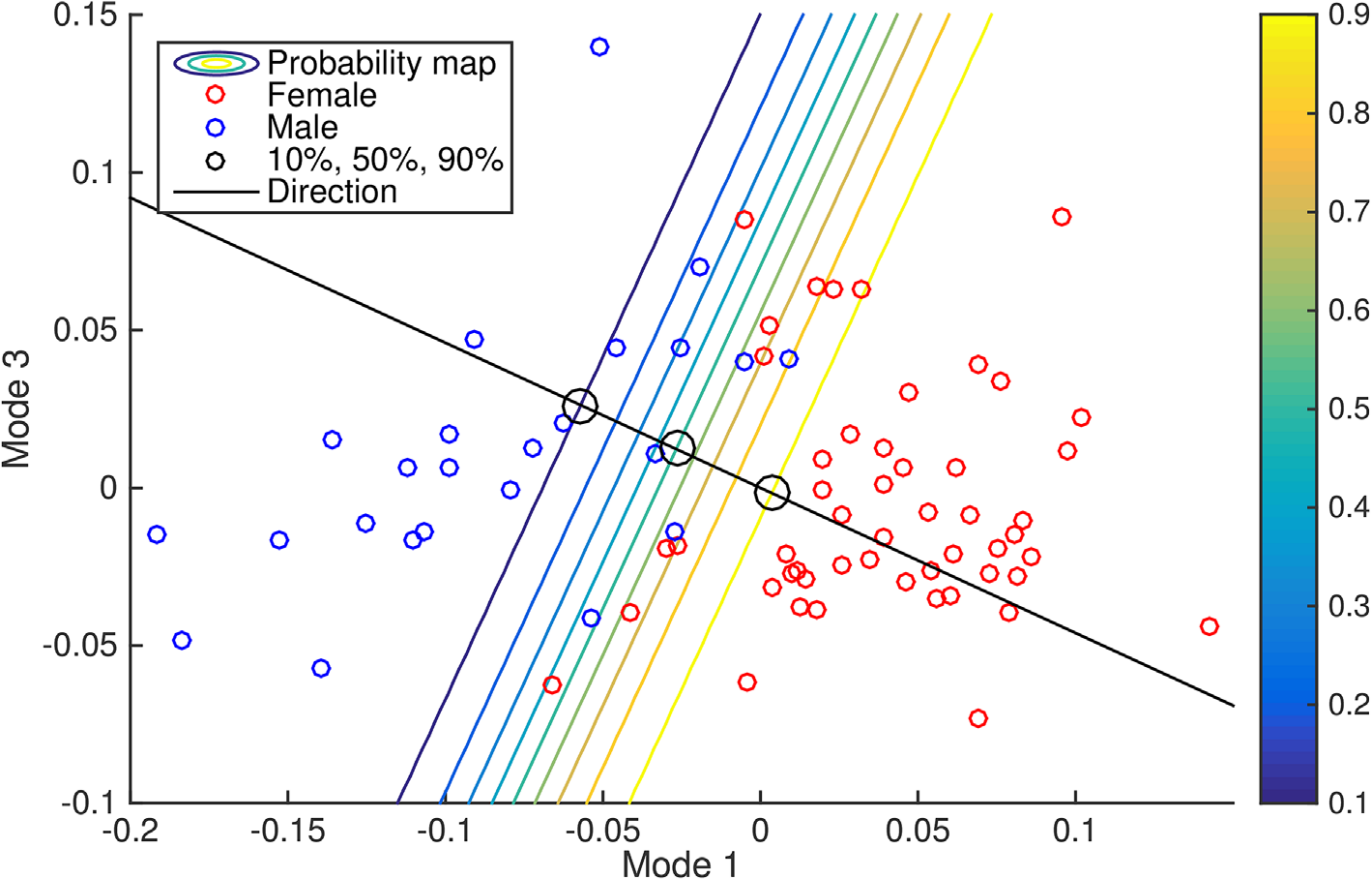
Visualization of discriminating direction. Scatter plot of coefficients for mode 1 and 3 showing male and female patients. The derived discriminating direction found by logistic regression and a contour plot showing the probability of a patient being female are shown.

**FIGURE 3:**
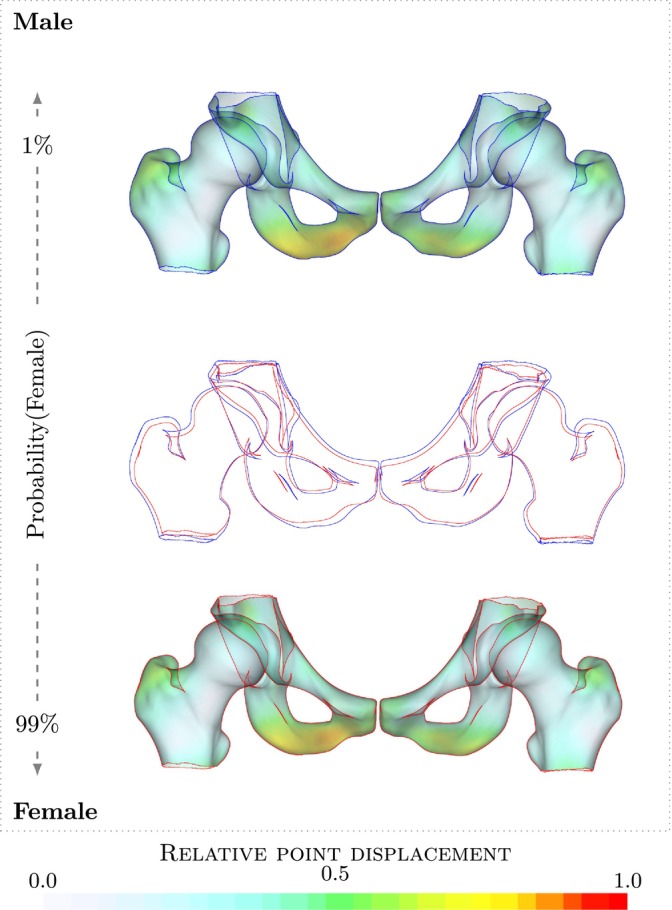
Sex difference visualization. Visualization of difference between male and female pelvis and femur model as described by the discriminating direction found by logistic regression. An overlay of extracted contours is shown with the female (red) and male (blue). An difference in the shape of the pubic arch can be seen.

The left and right hip models are shown in Figures 2 and 3, respectively. Each model is shown with the four most significant modes. Similar to the previous complete pelvis and femur model, the largest variation in the first mode is a difference in thickness of the pelvis and femur bones and the angle of the pubic bones. In addition, a clear difference in shape of the femur is shown and the acetabulum version is changed. In other modes, variation describes changes in neck‐shaft angle and in femoral coverage. In addition, it is apparent that there is a difference in head shape and subluxation of the femur.

### 3.2 Sex differentiation

**FIGURE 4:**
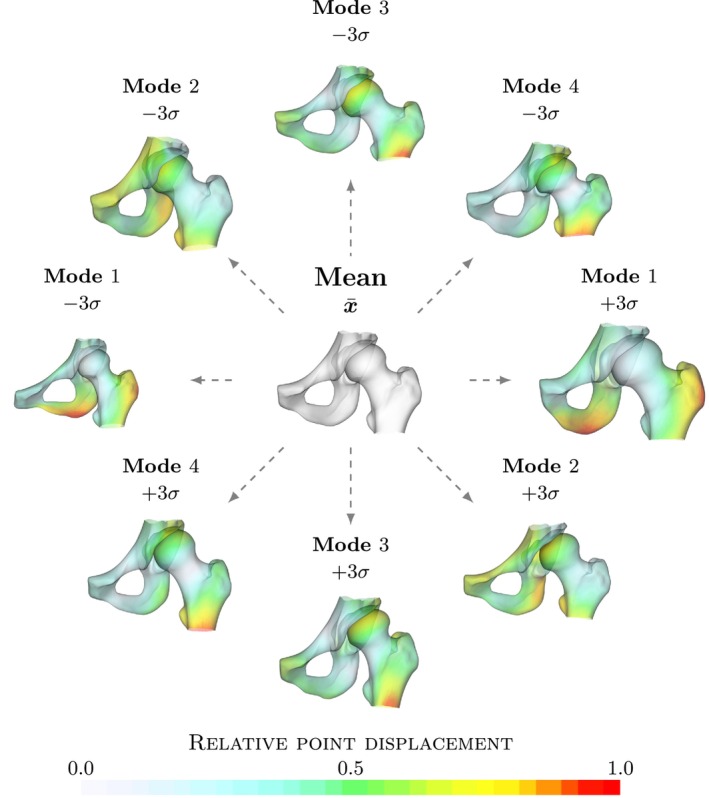
Visualization of left model. Visualization of the mean left pelvic bone and femur model and the four most significant modes explaining 67% of the total variation in the model. Each mode is shown as $\bar{x}$ ± 3 standard deviations. Colors indicate the point displacement normalized by the maximum displacement for a mode.

**FIGURE 5:**
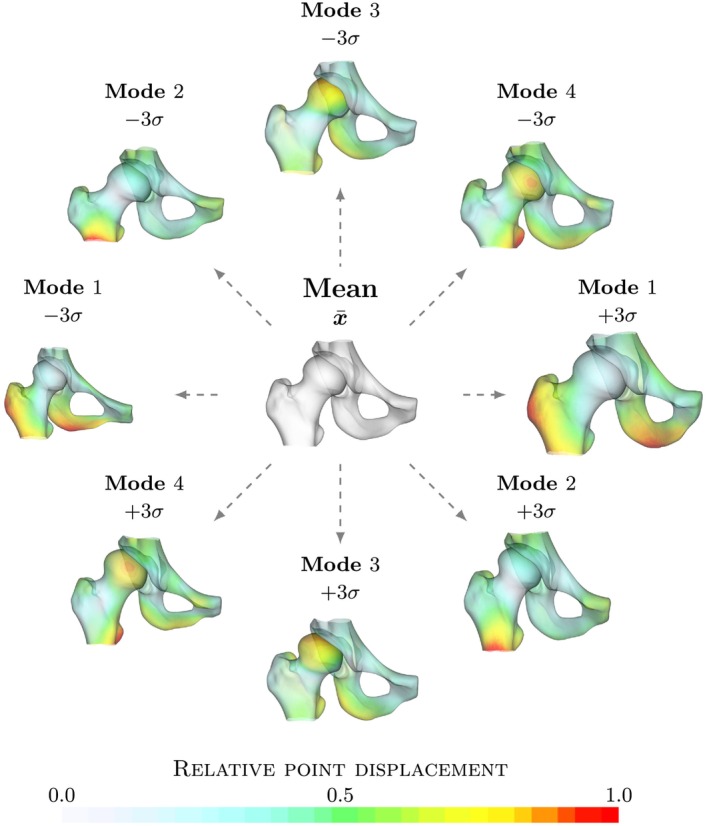
Visualization of right model. Visualization of the mean right pelvic bone and femur model and the four most significant modes explaining 66% of the total variation in the model. Each mode is shown as $\bar{x}$ ± 3 standard deviations. Colors indicate the point displacement normalized by the maximum displacement for a mode.

In the first regression experiment we will demonstrate the use of the complete pelvis and femur model to differentiate between sex and male and female pelvis shape and visualize the characteristic differences. To illustrate the interpretation of the discriminating direction found by logistic regression, we first create a regression model using just two modes. The scatter plot and direction discriminating between sex are shown in Figure 4. It is apparent that the derived direction optimally separates the patients by sex. The large circles indicate the points along the regression line through the mean shape associated with a probability of 10%, 50%, and 90% probability of being female. In Figure 5, we show the full model discriminating between sex using 28 modes which explain 95% of the total variance in the model. The model is visualized as 1% female and 99% female and an overlay of the outlines of the two is shown. The main differences between sexes are visible in the characteristic difference in shape of the pubic arch. There is also a small difference in the size between sexes, with the male model being slightly larger than females and a difference in shape of the ischium highlighted by a relative point displacement of more than 0.75. In leave‐one‐out cross validation, we found an area under the curve of 0.99 for predicting sex based on the logistic regression model, showing that the model has excellent predictive capabilities.

### 3.3 Hip dysplasia

**FIGURE 6:**
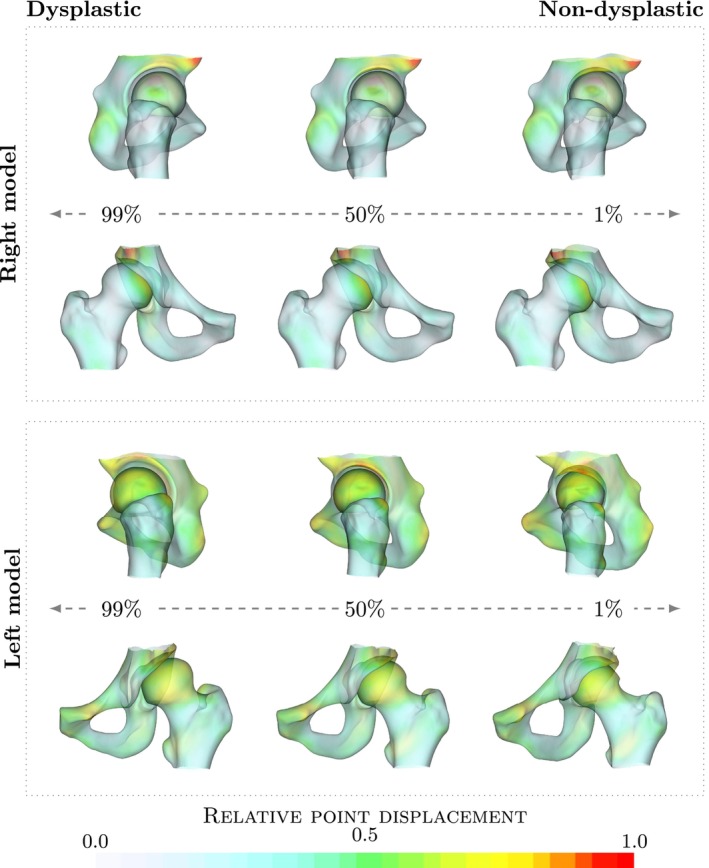
Visualization of dysplastic variation. Visualization of difference between dysplastic and non‐dysplastic hips as described by the discriminating direction found by logistic regression for the right (top) and left (bottom) models. For each model we show the lateral and anterior view. Colors indicate the point displacement normalized by the maximum displacement for a mode.

To investigate the relationship between hip dysplasia and shape, we used the individual left and right pelvic bone and femur models and performed logistic regression with presence/absence of hip dysplasia as outcome variable. The resulting shape changes relating to hip dysplasia are visualized in Figure 6. The depicted variation is characteristic of hip dysplasia, showing a transition from well covered femur to a steep roof. A difference in head shape and position of the head center can also be seen. In the dysplastic hip, the head loses the spherical shape and subluxation is found. This is especially visible in the left model, where the femur moves laterally and the change is more pronounced. Leave‐one‐out experiments predicting hip dysplasia resulted in an area under the curve of 0.73 for the left model and 0.84 for the right model.

### 3.4 Diagnostic angles

**FIGURE 7:**
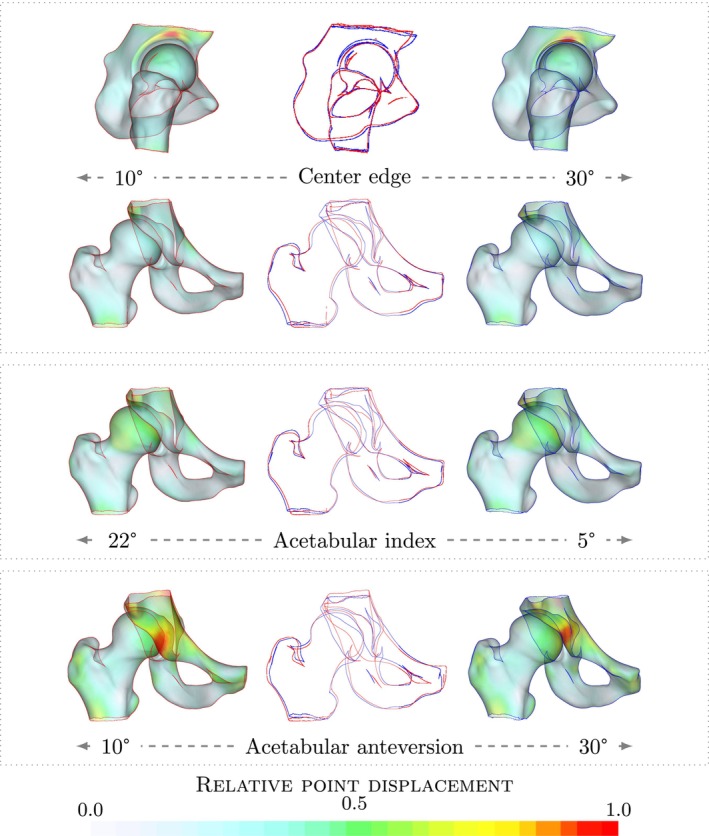
Visualization of diagnostic angles. Visualization of the shape variation associated with the regression line found by linear regression to predict diagnostic angle measurements. Colors indicate the point displacement normalized by the maximum displacement.

We demonstrate the ability to use linear regression to determine angle measurements and extract the shape variation associated with varying angle measurements. In Figure 7, we show the associated variation for different angle measurements for the right hip. For the CE angle we see that points along the lateral acetabular rim are highlighted in the lateral view. In the anterior view, it is apparent that the coverage of the femur increases with increasing CE angle. The overlay shows that the main differences are present at the lateral‐superior edge of the acetabulum and the position of the head. The center of the femoral head moves medially for an increasing CE angle. Similar variation is observed for the acetabular index angle. To study the morphological variation with respect to the version of the acetabulum, we investigated the relationship between the AcAV angle and the shape model. A strong variation along the anterior edge of the acetabulum is shown in Figure 7.

Increasing acetabular anteversion results in less anterior coverage of the femur. Decreasing acetabular anteversion results in the cross‐over of the lateral edge of the anterior and posterior walls of the acetabulum as well as the appearance of the prominent ischial spine sign. In the overlay of the two models, it is apparent that the anterior edge is shifted laterally along the whole height of the acetabulum, with the lines remaining approximately parallel. This is in contrast to the variation observed for the center‐edge angle and acetabular index angle, for which the change is mainly located in the superior aspect of the acetabulum. This suggests that the version influences the complete acetabulum and not only the superior aspect of the acetabulum.

**FIGURE 8:**
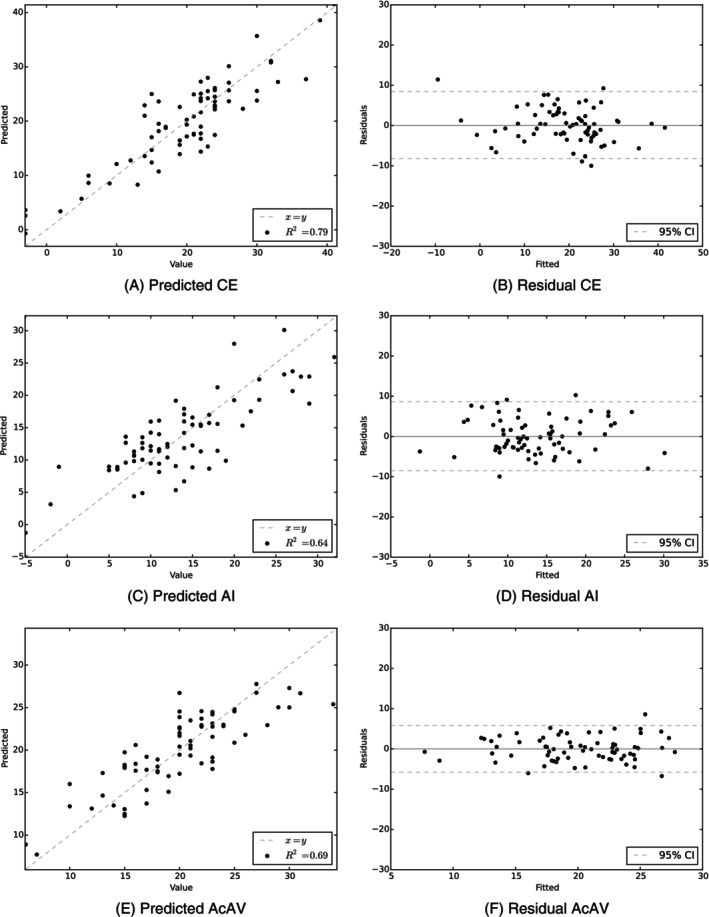
Predicted and residuals for diagnostic angles. Linear regression results predicting angle measurements using the right hip model in leave‐one‐out experiments. Graphs of predicted values and residuals for each angle measurement are shown.

In Figure 8, we show the results for the leave‐one‐out cross‐validation experiments predicting angle measurements based on shape parameters. We find that angle measurements can be predicted within a 95% confidence interval of 10° for the center‐edge and acetabular index angle. For the acetabular index, the angle can be predicted within a 95% confidence interval of approximately 5°. The small confidence interval may be due to the significant association between sex and the acetabular anteversion angle and the small standard deviation of the angle.

## 4 DISCUSSION

In this work, we presented combined statistical shape models of the symptomatic pelvis and femur. The main modes of variation describe both the difference in shape due to sex and the varying degrees of hip dysplasia. Using logistic regression, we demonstrated that the statistical shape models can be used to differentiate between sex and both dysplastic and non‐dysplastic hips with an area under the curve of 0.99 and ≥0.73, respectively. In addition, we showed that the resulting regression model can be used to visualize the shape variation associated with the predicted dependent variables such as hip dysplasia, sex, and different angle measurements. We found that the variation associated with sex described by the combined model was in accordance with previous findings by Decker et al. (2011). The most prominent difference was the difference in the thickness of the pelvic bones and the shape of the pubic arch between males and females. In females, the width of the pubic opening was observed to be larger than in males. This difference may be attributed to the extra space needed for the passage of the fetus during childbirth. The shape of the pubic arch was also different between males and females, with males having a smaller angle similar to the findings of Decker et al. (2011). The femur was larger in males than in females.

We found that the variation associated with hip dysplasia was in agreement with the typical description of the dysplastic acetabulum. The roof was steep and the coverage of the femur was incomplete. We found that the shape of the femoral head was less spherical with increasing probability of hip dysplasia. Analysis of manual angle measurements showed that the coverage of the dysplastic hip was significantly reduced globally, in agreement with the findings of Mechlenburg et al. (2004).

An interesting clinical observation is that we found that the female acetabulum is significantly more anteverted than in males. Furthermore, we found that reduced anteversion resulted in the appearance of the cross‐over sign and the prominent ischial spine sign as described by Fujii et al. (2011) in a study of retroversion. Similar to previous studies (Fujii et al., 2011; Kalberer et al., 2008), we find that the version of the acetabulum affects the whole acetabulum and is not limited to the superior aspect. This supports the suggestion by Kalberer et al. (2008), that appearance of the prominent ischial spine is a result of the complete rotation of the acetabulum. Larson et al. (2015) also found that the cross‐over sign and posterior wall sign were a frequent finding and were more common in males than in females in an asymptomatic cohort. They concluded that retroversion might be a normal variation. In a study by Steppacher et al. (2014), they investigated the difference in shape of the lunate surface in patients with various hip diseases. They also found similar results and concluded that the retroversion of the acetabulum was the result of malorientated acetabulum. This is in further agreement with a previous two‐dimensional study using radiographs (Jamali et al., 2007).

We found no association between hip dysplasia and acetabular anteversion. We therefore believe that the version of the acetabulum is a separate morphological variation independent of hip dysplasia. This finding is also supported by the fact that we find similar values for the acetabular anteversion angle for males and females as in normal measurements (Mechlenburg et al., 2019; Tallroth & Lepistö, 2006) as well as in young patients with hip pain (Hetsroni et al., 2013). The clinical relevance of this finding may have important implications for the treatment of patients with hip dysplasia. More specifically, it may be important to consider the amount of correction applied for retroversion of the acetabulum when performing periacetabular osteotomies on male patients. Taken together, our findings suggest that there may be an interesting explanation for the difference in observed incidence of hip dysplasia and FAIS between sex. As increased retroversion is associated with increased risk of impingement (Ganz et al., 2003) and males are more retroverted, it may be plausible that together they result in an increased incidence of FAIS in males. Conversely, the increased incidence of hip dysplasia in females with a deficient anterior coverage may be related to the increased anteversion of the acetabulum in females. To our knowledge, no previous study has shown the relationship between regression coefficients and the shape parameters of the statistical shape model for visualization purposes. An interesting finding was that the resulting models showed that the relative point displacement was spatially localized with areas associated with the variation. For example, the largest relative point displacements were found to be along the acetabular rim in both hip dysplasia and changes in center‐edge and acetabular index angles. Although intuitive in our study, this method may be used in studies of other anatomical shapes where the link may be less clear. A clear advantage of our method is that no distance metric has to be defined and the optimization of linear regression models is fast and relatively easy with standard software. A limitation of the current study was that no normal volunteers were included in the model, but only the contralateral side of patients with unilateral hip dysplasia. However, as the angle measurements in the normal hips were consistent with previous studies of the normal angles (Mechlenburg et al., 2019; Tallroth & Lepistö, 2006), we believe our normal hips are representative for normal hips. During the model building the femur position was standardized in order to remove variation due to the difference in pose of the femur. However, the difference in pose of the femur may also play a role in sex and disease morphological differences. It is also known that the femur is more anteverted in females (Hetsroni et al., 2013). Therefore, future investigations may develop methods to include both the shape and pose variability of the femur. Another possible direction for future work may be to use the shape model created in this study as a starting point to create patient specific three‐dimensional models from pre‐operative x‐rays or intra‐operative fluoroscopy images using a method as described by Zheng et al. (2009). Since the current model was created based on patients with hip dysplasia, the model may be better suited to reconstruct the anatomy of the dysplastic patient. This would eliminate the need for pre‐operative CT scans for use with surgical guidance systems and reduce the radiation dose to the patient.

## 5 CONCLUSIONS

In conclusion, we introduced a simple method to visualize the relationship between the morphology of the hip and its relationship to both hip dysplasia and sex differences. Our findings suggest that retroversion is a result of decreased anteversion of the acetabulum and is primarily associated with sex. This finding is of clinical relevance and should be taken into account during the reorientation of the acetabulum in periacetabular osteotomies.

## Supporting information


**Data S1** Supporting Information.
