# Supplementary material for: Morphology of the dysplastic hip and the relationship with sex and acetabular version
Source: Clin Anat. 2024 May 8;38(5):512–22. doi: 10.1002/ca.24174 (PMC12163104; doi:10.1002/ca.24174)
Supplement: Supplementary file 1 — Data S1 Supporting Information. [file CA-38-512-s001.docx]

**Supplements**

**1 Point distribution model**

The PDM can be constructed using the set of aligned shapes obtained from the previous steps. Each of the N shapes with n points is represented as a vector of 3n concatenated point coordinates such that the i^th^ shape is represented as *x_i_* = [*x_i,_*_1_*,y_i,_*_1_*,z_i,_*_1_*,...,x_i,n_,y_i,n_,z_i,n_*]. The mean shape can then be calculated as:

The deviation of individual shapes from the mean can then be quantified as *d****x****_i_* = ***x****_i_ −* ***x*¯**. The displacement vectors dxi represent the displacement of individual points from the mean shape for each shape and can be used to calculate the covariance matrix C defined as:

**

Performing eigen analysis of the covariance matrix, results in the principal components, the main modes of variation of the included shapes around the mean. Any shape x can be represented as a linear combination of the mean shape x̅ and a weighted combination of these modes of variation:

where Φ is the matrix of eigenvectors and b are the shape parameters that weigh the contribution of each eigenvector.

Varying the shape parameters between appropriate limits, e.g. 2 standard deviations, results in plausible variation described by the statistical shape model.

**2 Regression analysis**

We use regression models to find the characteristic difference in shape associated with dependent variables such as sex, dysplasia, or angle measurements. In this section, we derive an expression in order to visualize the characteristic shape differences using the resulting models. For categorical dependent variables (sex, dysplasia), the probability P associated with the dependent variable Y is formulated as a logistic function:

based on a given set of shape parameters b = [b1, ..., bt] and regression coefficients β = [β0, ..., βt]. Note, that the shape parameters could be the point coordinates of aligned shapes, or the coordinates of a fitted shape model with t modes. y is a binary indicator for the dependent variable, where for example y = 1 for female and y = 0 for male. A regression model can then be found by optimizing:

where the first term is the likelihood of the model and the second term provides regularization to constrain the size of the weights ^1^ and j is an indicator for the dependent variable. The regularization is known as ridge regression and is based on the L2 norm ^2^. The strength of the regularization term is determined by λ which is found by leave-one-out or generalized cross-validation ^3, 4^. After optimization of the logistic regression model, the values of the regression coefficients β determine the direction of changes in the independent variables (the shape) associated with the dependent (outcome) variable, where β0 is the intercept term and βi is the regression coefficient associated with mode i for i > 0. The intercept term is the log of the odds associated with the prevalence of the dependent variable. To visualize the characteristic shape differences related to the outcome Y, we choose to visualize the mean shape deformed along the discriminating direction. Selecting a point on the regression line results in shapes describing the characteristic shape associated with a certain value of probability P. We now rewrite the logistic function in terms of the logit function, such that we obtain:

where for brevity we have introduced *p*= P (Y = 1|β, b). The resulting expression is a linear function of the shape parameters and regression coefficients. For visualization, we select the point b = cβ, where c is a scaling factor determining the position on the regression line. Rearranging we obtain:

Now we substitute the cβi for bi, where c is the scaling parameter to be determined and obtain:

which can subsequently be rewritten as:

to obtain an expression for c for a given probability p and β.

For linear regression, a similar expression can be derived as:

With the derived regression parameters and scaling parameters, we can calculate the characteristic shapes as

$$\boldsymbol{x}=\bar{\boldsymbol{x}}+c\Phi\boldsymbol{\beta}$$

**3 Model building specifications**

Segmentations were performed using the automatic graph cut segmentation method described in ^5^. Masks for image registration were created by dilating the segmentation by 5 voxels and excluding areas of the adjacent bone. Soft-masks were created using the Q function with a mean of 0 and standard deviation of 1. All images in the registration were cropped to contain the bone of interest including a margin of 10 voxels on all sides. The individual pairwise registrations consisted of three steps. Initial alignment was established with a rigid and affine transformation using only the soft-masks. Subsequently, a non-rigid B-spline registration was performed with both the CT grey-scale images and the masks. A final non-rigid B-spline registration using only the soft-masks was performed to refine the alignment of the contours of the bones. All registrations were performed using the sum of squared differences similarity metric and with Elastix 4.7 ^6^. Each registration was performed on three levels with a Gaussian pyramid with smoothing and down-sampling ^7^. Final grid-spacing for the initial grey-scale B-spline registration was 10 mm and 5 mm for the final soft-mask registration. The inverse transformation was calculated with a final grid-spacing of 5 mm. The mean bone shapes were extracted, smoothed and simplified to approximately 10,0000 points using Visualization Toolkit ^8^. The alignment of the pelvis was performed and the femur pose was corrected.

The pose correction converged to within machine precision in 5 iterations. First, a combined model of the complete pelvis and femur was constructed. This model preserves the orientation between the left and right pelvic bones. In addition, two models of the individual left and right pelvic bones and femur were constructed. The PDM models were created using a custom application based on the open source framework for statistical shape modeling Statismo ^9^. Finally, the regression models were created. Due reduce the dimensionality of the model, we retained the number of modes that explain 95% of the variation of the model and performed regularization during the optimization of the regression models. We used the L2 norm (ridge regression) for regularization for all regression experiments. The regularization parameter λ was determined by 10 fold cross-validation with 100 values evenly spaced on the log scale. The minimum and maximum values were selected to ensure that the global optimum was found by visual inspection of the deviance versus λ plot. For logistic regressions, a stratified cross-validation was performed and the criterion to minimize was the negative log-likelihood. For linear regression, the R2 coefficient of determination was used as cross-validation metric. Each regression model was evaluated using a leave-one-out cross-validation (optimizing regularization parameters excluding the tested shape), in order to obtain a true estimate of the predictive value of the model. All regression models were created with Scikit-learn.

**References**

1. Hoerl AE, Kennard RW. Ridge Regression: Biased Estimation for Nonorthogonal

Problems. Technometrics. 1970;12(1):55{67.

2. Golub GH, Hansen PC, O'Leary DP. Tikhonov Regularization and Total Least

Squares. SIAM J Matrix Anal & Appl. 1999;21(1):185{194.

3. Golub GH, Heath M, Wahba G. Generalized Cross-Validation as a Method for

Choosing a Good Ridge Parameter. Technometrics. 1979;21(2):215{223.

4. de Bruijne M, Lund MT, Tank_o LB, Pettersen PC, Nielsen M. Quantitative

vertebral morphometry using neighbor-conditional shape models. Med Image

Anal. 2007;11(5):503{512.

5. Raedt Sd, Mechlenburg I, Stilling M, Rømer L, Søballe K, de Bruijne M. Automated measurement of diagnostic angles for hip dysplasia. Proceedings of SPIE - The International Society for Optical Engineering. 8670. 09-. 10.1117/12.2007599.

6. Klein S, Staring M, Murphy K, Viergever MA, Pluim JPW. elastix: a toolbox for

intensity-based medical image registration. IEEE Trans Med Imaging. 2010;29(1):196{205.

7. Shamonin DP, Bron EE, Lelieveldt BPF, Smits M, Klein S, Staring M, et al. Fast

parallel image registration on CPU and GPU for diagnostic classification of

Alzheimer's disease. Front Neuroinform. 2013;7:50.

8. Martin K, Lorensen B. The Visualization Toolkit. An Object-oriented Approach

to 3D Graphics. Ingram; 2006.

9. Albrecht T, Gass T, Goksel O, Luthi M. Statismo-A framework for PCA based statistical models. Insight. 2012;.
